# VDR gene TaqI (rs731236) polymorphism affects gut microbiota diversity and composition in a Caucasian population

**DOI:** 10.3389/fnut.2024.1423472

**Published:** 2024-09-12

**Authors:** Rocío Gonzalez-Soltero, Mariangela Tabone, Mar Larrosa, Maria Bailen, Carlo Bressa

**Affiliations:** ^1^Masmicrobiota Group, Madrid, Spain; ^2^Faculty of Biomedical and Health Sciences, Universidad Europea de Madrid, Madrid, Spain; ^3^Department of Nutrition and Food Science, Faculty of Pharmacy, Universidad Complutense de Madrid, Madrid, Spain; ^4^Department of Preventive Medicine, Public Health and Microbiology, Faculty of Medicine, Universidad Autónoma de Madrid, Madrid, Spain; ^5^Faculty of Experimental Sciences, Universidad Francisco de Vitoria, Madrid, Spain

**Keywords:** VDR, microbiota, TaqI (rs731236), microbiome, polymorphism

## Abstract

**Background:**

The VDR gene is identified as a crucial host factor, influencing the gut microbiota. The current research focuses on an observational study that compares gut microbiota composition among individuals with different VDR gene TaqI polymorphisms in a Caucasian Spanish population. This study aims to elucidate the interplay between genetic variations in the VDR gene and the gut microbial composition.

**Methods:**

87 healthy participants (57 men, 30 women), aged 18 to 48 years, were examined. Anthropometric measures, body composition, and dietary habits were assessed. VDR gene polymorphism TaqI rs731236 was determined using TaqMan assays. The V3 and V4 regions of the 16S rRNA gene were sequenced to study bacterial composition, which was analyzed using QIIME2, DADA2 plugin, and PICRUSt2. Statistical analyses included tests for normal distribution, alpha/beta diversity, ADONIS, LEfSe, and DESeq2, with established significance thresholds.

**Results:**

No significant differences in body composition or dietary habits were observed based on VDR genotypes. Dietary intake analysis revealed no variations in energy, macronutrients, or fiber among the different VDR genotypes. Fecal microbiota analysis indicated significant differences in alpha diversity as measured by Faith’s Phylogenetic Diversity index. Differential abundance analysis identified taxonomic disparities, notably in the genera *Parabacteroides* and *Butyricimonas*.

**Conclusion:**

Overall, this study suggests potential associations between genetic variations in the VDR gene and the composition and function of gut microbiota.

## 1 Introduction

The Vitamin D receptor (VDR) is a conserved nuclear receptor controlling the primary physiological actions vitamin D’s, active form, 1α,25-dihydroxyvitamin D3 (1,25(OH)2D3) (1). It is part of the ligand-responsive transcription factors in the nuclear hormone receptor superfamily (2). This receptor is prominently expressed in multiple tissues, including the intestines, adipose tissue, liver, and immune cells, with higher expression observed in small intestine and colon epithelial cells (3, 4). It plays a key role in regulating metabolic and immune functions, serving as an essential regulator of intestinal cell proliferation and differentiation, barrier function, intestinal immunity and host-microbial interactions (5).

The *VDR* gene exhibits an extensive array of allelic variants within the *VDR* locus, encompassing over 900 variants. Among these, the most extensively researched polymorphisms include ApaI (rs7975232), BsmI (rs1544410), TaqI (rs731236), and FokI (rs10735810) (6). Regarding the BsmI–ApaI–TaqI polymorphism, the findings remain controversial. Although there is a tendency for the Bat haplotype (C allele for TaqI) to exhibit higher levels of mRNA expression compared to the baT haplotype (T allele for TaqI), the results have been inconsistent. Nevertheless, there appears to be a general trend indicating that the Bat haplotype demonstrates a more favorable response than the baT haplotype (7). The TaqI restriction site polymorphism (T/C) has been linked with health status in various studies. The T allele of the TaqI polymorphism has been associated with the risk of suffering post-transplant diabetes mellitus (DM) (8), and osteoarthritis in the knee (9). Links have been established between TaqI polymorphisms and susceptibility to Crohn’s disease, multiple sclerosis or primary hyperparathyroidism, renal carcinoma, and the risk of metastases in breast cancer (10). However, controversial results have been observed between TaqI polymorphisms and prostate cancer, DM type 2 and nephrolithiasis (10). Additionally, *VDR* has been proposed as a potential key player in the pathogenesis of obesity, through the discovery of a strong association between the *VDR* TaqI ‘T’ allele and obesity in a Greek population (11). A study in Iranian population also found a significant association between the TaqI ‘T’ allele and obesity, contributing to an increment in the BMI of 3 kg/m^2^ per risk allele (12), whereas in Asian Indians the tt (CC) genotype of the TaqI polymorphism was associated with ≥5% weight loss after lifestyle interventions (13). However, no significant relationships were observed between TaqI genotypes when analyzed as a risk score in a Mendelian Randomization study in 42,024 Caucasians. A significant causal effect of the vitamin D metabolism-risk score on obesity was not found (14). The combined effects of obesity and VDR gene polymorphisms on susceptibility to type 2 diabetes mellitus (T2DM) have been explored. However, the specific relationship between TaqI polymorphism and T2DM remains unclear (15). Overall, the association between TaqI VDR polymorphism and obesity or T2DM is still under investigation, and further research is required to fully understand the relationship. However, these data suggest that lifestyle interventions could reveal one or several modifiable factors associated with the TT genotype that may be responsible for the effect.

The human *VDR* gene is the first to be identified in a genome-wide association analysis, involving two cohorts, totaling 1812 individuals, as a critical host factor that shapes the gut microbiota at the genetic level (16). In mice lacking the *VDR* gene, significant shifts in the microbiota relative to control mice have been observed. These changes encompass reduced levels of bacteria belonging to the *Firmicutes* phylum, increased levels from the *Bacteroidota* and *Proteobacteria* phyla (17), modifications in various bacterial genera, including *Eubacterium*, *Bacteroides*, and *Salmonella* (18), and shifts in critical pathways within the intestinal microbiota. These alterations may have implications for detoxification, infection, cancer, and other diseases (19). The absence of VDR leads to dysbiosis, indicating a critical role of VDR in shaping gut microbiota communities (20).

In this work we have conducted an observational study to compare the gut microbiota composition among individuals with different VDR gene TaqI (rs731236) polymorphisms, in a Caucasian Spanish population. By analyzing genetic variations in the VDR gene and their potential impact on the gut microbial composition, we aim to shed light on the interplay between these factors and contribute to the development of personalized medical approaches.

## 2 Materials and methods

### 2.1 Ethics approval and consent to participate

The study was an observational study conducted in accordance with the Declaration of Helsinki, and the protocol received approval from the Research Ethics Committee of the Community of Madrid (CEIm-R; Ref: 47/560280.9/18). All participants provided written informed consent.

### 2.2 Participant characteristics

The study included a total of 87 healthy participants, comprising 57 men and 30 women, aged 18 to 48 years. Inclusion criteria included healthy Caucasian men and women aged 18–50 years with a body mass index (BMI) of 18.5–25 kg/m^2^ and exclusion criteria any kind of pathology (current or within 6 months prior to the study), previous gastrointestinal surgery, antibiotic intake 3 months before the study, smoking, use of prebiotics, probiotics, or nutritional complements, being vegetarian or vegan diets, and pregnancy or lactation.

### 2.3 Anthropometry and body composition

Height and weight were measured with a tallimeter (Asimed T2, Barcelona, Spain) and a balance scale (Ano Sayol SL, Barcelona, Spain), respectively. Body mass index (BMI) was calculated as weight (kg) divided by height (m^2^). Body composition, including estimated visceral adipose tissue (VAT), body fat percentage (BFP), and body fat mass (BFM), was evaluated on the day of stool sample collection using dual-energy X-ray absorptiometry (DEXA; Hologic DEXA scan, Hologic Inc., Barcelona, Spain).

### 2.4 Dietary habits

A self-reported food frequency questionnaire (FFQ), validated for the Spanish adult population, was used to gather information on the frequency and quantity of food consumption. The questionnaire includes 93 items encompassing various foods and food groups. Participants reported their usual intake of different foods and beverages categorizing their consumption frequency within predefined intervals (never or less than once a month, 1–3 times per month, 1 time per week, 2–4 times per week, 5–6 times per week, 1 time per day, 2–3 times per day, 4–5 times per day, 6 or more times per day). The data collected from the questionnaire was input into the DietSource 3.0 software (Novartis, Barcelona, Spain), a dietary analysis program to calculate the intake of macronutrients, fiber and total energy (21).

### 2.5 Sample collection

Participants were provided with the Fe-Col^®^ Fecal Sample Collection Kit (Alpha Laboratories, Hampshire, United Kingdom), along with an insulated bag and ice blocks to preserve the samples until delivery to the laboratory. Stool samples were stored at −80°C until extraction.

### 2.6 DNA extraction

DNA from humans and bacteria was extracted from 100 mg of stool sample using the commercial E.Z.N.A.^®^ Stool DNA Kit (Omega Biotek, Norcross, GA) and a bead-beating homogenizer (Bullet Blender Storm, Next Advance, NY). The concentration and purity of DNA were assessed using the Quant-iT PicoGreen dsDNA Assay Kit (ThermoFisher Scientific, Waltham, MA) and an FP-8300 spectrofluorimeter (Jasco, Tokyo, Japan). Bacterial DNA was utilized for microbiota analysis, while human DNA was used for *VDR* genotyping.

### 2.7 VDR genotyping

Applied Biosystems TaqMan^®^ SNP Genotyping Assays (Applied Biosystems, Foster City, CA, Country; assay ID: C_2404008_10) were used for allelic discrimination analysis of the TaqI VDR (rs731236) gene polymorphism. The StepOnePlus Real-Time PCR system (ThermoFisher Scientific) was employed following a protocol that included denaturation at 95°C for 10 min, then 50 cycles of denaturation at 92°C for 15 s, annealing/extension at 60°C for 1 min, and a final extension step of 30 s at 60°C. Fluorescence analysis was performed with Allelic Discrimination 7,500 software v.2.0.2. After genotyping, participants were classified based on the VDR genotype: TT, TC and CC for subsequent analyses. The allele and genotypes frequencies were calculated using the SNPStat program (22). Experiments were conducted three times to solve incongruences in the genotype assignment. In cases where conflict arise, the Allelic Discrimination 7,500 software v.2.0.2 provided by Thermofisher was used.

### 2.8 Sequencing and bioinformatics

The hypervariable V3 and V4 regions of the 16S rRNA gene were amplified using the primer pair 5’-TCGTCGGCAGCGTCAGATGTGTATAAGAGACAG-3′ and 5’-GTCTCGTGGGCTCGG AGATGTGTATAAGAGACAG-3′. The 459 bp amplicon was visualized in a 0.8% agarose gel stained with ethidium bromide, and bands were cut and purified using the MinElute Gel Extraction Kit (Qiagen, Hilden, Germany). The DNA amplicons were sequenced on a MiSeq Illumina platform (Illumina, San Diego, CA). The sequencing outputs were analyzed using the Quantitative Insights into Microbial Ecology (QIIME2) program, version V2023.2 (23). The 16 s rRNA paired reads were imported in QIIME2 and processed with the DADA2 plugin (24), with the maximum expected error threshold set to 2.0 for both forward and reverse reads. Taxonomy assignments were conducted using the classify-sklearn method (25) and a customized classifier based on the SILVA reference database (26, 27). To build the customized reference database, sequences, aligned with our primers (forward primer sequence: CCTACGGGNGGCWGCAG, reverse primer sequence: GACTACHVGGGTA TCTAATCC) were extracted from the SILVA 138.1 database clustered at 99% identity. The classifier was trained using our tailored reference reads and SILVA 7-levels for reference taxonomy, including species probabilities (weights) likely observed in human stool^1^ (28, 29). Diversity analyses were performed through QIIME 2’s q2-diversity plugin. Beta-diversity was assessed by calculating the Bray-Curtis, Jaccard, unweighted and weighted Unifrac distance metrics. For alpha-diversity, observed features (ASVs), Evenness, Shannon and Faith’s Phylogenetic Diversity indices were calculated. Kyoto Encyclopedia of Genes and Genomes (KEGG) ortholog abundances predictions were obtained with the Phylogenetic Investigation of Communities by Reconstruction of Unobserved States (PICRUSt2) software (30) using default “max parsimony” method for hidden-state prediction and a Nearest Sequenced Taxon Index (NSTI) value of 2.0.

### 2.9 Statistical analysis

Statistical analysis was conducted using QIIME2 v2023.2, SPSS software v26.0 (SPSS, Chicago, IL) and the R statistical package v4.1.1 incorporating the packages: Phyloseq (31) and microbiomeMarker (32). The Shapiro–Wilk test was used to assess the normal distribution of variables; non-parametric tests were performed when a normal distribution was not observed. Differences between groups in terms of alpha-diversity were assessed using the Kruskal–Wallis test (33). A significance threshold of adjusted *p* value = 0.05 was applied in all calculations. For beta-diversity, the differences between groups were assessed using the ADONIS permutation-based statistical test adjusted for sex (34). A significance threshold of p value = 0.05 was used in all calculations. Linear discriminant analysis coupled with effect size (LEfSe v1.0) was employed to identify bacterial-associated pathways that exhibited differential representation between groups, utilizing default settings. Initially, the Kruskal-Wallis rank test was applied to discern specific differences among groups. Subsequently, the Wilcoxon rank test was conducted, utilizing sex as subgroups, to assess the consistency of differences identified in the preceding step. Significance was set at *p* < 0.05. To determine whether the allele frequencies were in Hardy–Weinberg equilibrium for the *VDR* genotypes we employed the Chi-square (χ^2^) test. The determination of differentially abundant bacterial taxa between VDR gene TaqI polymorphisms was performed using analysis of composition of microbiomes (ANCOM-BC) (35) and DESeq2 (36) adjusting for sex. DESeq2 applies a negative binomial generalized linear model for each taxon count, estimating log-fold changes between two classes and using a Wald test on this value for significance testing. ANCOM-BC addresses variability in microbiota samples by assuming that the observed sample represent an unknown fraction of the ecosystem’s unit volume, with varying sampling fractions among samples. This method includes sample-specific offsets in a linear regression framework, estimated from observed data, serving as bias corrections. Both DESeq2 and LEfSe analyses were conducted on prefiltered data. The raw dataset was prefiltered to remove rare taxa occurring in less than 20% of the samples. ANCOM-BC being more conservative and precise identifies only a small number of ASVs as significant, potentially reducing sensitivity. Therefore, DESeq2 was also utilized to enhance sensitivity (37). The *p*-values from the DESeq2 test were adjusted for multiple testing using the Benjamini-Hochberg false discovery rate (FDR) procedure, with results deemed significant at FDR <0.05, in line with DESeq2’s default settings. The p-values from the ANCOM-BC test were adjusted using the Holm–Bonferroni method, with results considered significant at alpha <0.05, as per ANCOMBC’s default settings.

## 3 Results

### 3.1 Subjects, genotypes and allelic frequencies

Eighty-seven subjects were recruited for this study comprising 57 men and 30 women. Genotyping results for the rs731236 (TaqI) polymorphism were obtained and are presented in Table 1. Compared to the 1,000 Genomes Project reference the calculated allele frequencies (T = 0.61 and C = 0.39) matched those expected for a European population sample. When examining genotypes, TT homozygotes were found at a frequency of 0.43, while TC heterozygotes and CC homozygotes had frequencies of 0.38 and 0.20, respectively, in accordance with Hardy–Weinberg equilibrium (*p* = 0.071; Table 1).

**Table 1:** SNP allele and genotype frequencies.

rs731236 (TaqI, C/T)		SNP allele frequencies (*n* = 87)	Genotypes	Frequencies*
T		0.39	TT	0.43
C		0.61	TC	0.38
			CC	0.20

*Genotype frequencies were in Hardy–Weinberg equilibrium (*p* = 0.071) (21).

Furthermore, we grouped the carriers for the common allele (TT) for VDR and compared their frequency with carriers of the rare allele (CC and TC) across all the parameters of this study (body composition, dietary habits, and microbiota). Additionally, carriers of the recessive alleles (CC) were grouped and compared against carriers of the dominant allele (TT and TC) following the approach previously used by Vasilopoulos (11).

### 3.2 Body composition and dietary habits

In this study, we explored the potential impacts of the polymorphisms on body composition and dietary habits. The aim to control for dietary habits was to ensure that observed differences are not attributed to various dietary patterns, especially given the significant influence of diet on the microbiota. After categorizing participants based on their *VDR* genotypes (see Supplementary Table S1), no significant differences in body composition parameters were observed between the groups (genotypic or dominant and recessive allele grouping).

Furthermore, we utilized a Food Frequency Questionnaire (FFQ) to record and analyze the participants’ dietary intake, including energy, macronutrients, and fiber. Subsequent comparisons of various *VDR* genotypes (Supplementary Table S2) revealed no statistically significant variations in the intake levels of macronutrients (such as carbohydrates, protein, fat, protein/carbohydrate ratio, and protein/fat ratio), fiber, and total energy.

### 3.3 Fecal microbiota

**Figure 1:**
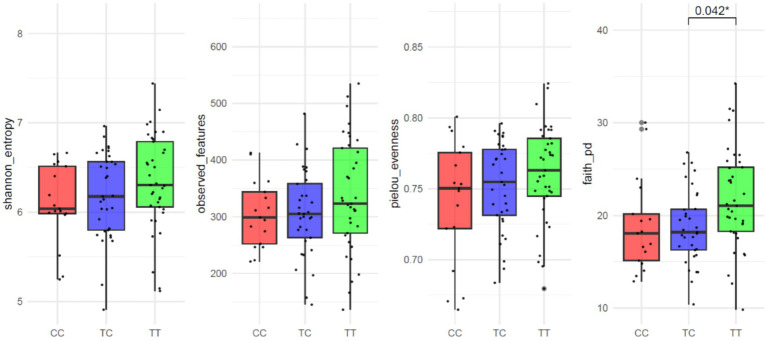
Alpha-diversity parameter of VDR polymorphisms: Shannon entropy, Observed features, Pielou evenness and Faith’s Phylogenetic Diversity.

The average number of reads per sample was 167,839. In the study of alpha diversity indices among *VDR* genotypes, no significant differences were observed in measures of richness or evenness except for Faith’s Phylogenetic Diversity index. This index, which accounts for both species richness and phylogenetic distance among species present in a community, showed significant differences (*p* = 0.013; Figure 1). The results for observed features (*p* = 0.250), Shannon entropy (*p* = 0.138) or Pielou evenness (*p* = 0.320) indicated no significant variations. Similarly, for β-diversity (Supplementary Figure S1), no significant differences were detected across the following distance metrics: Bray–Curtis (R^2^ = 0.02 *p* = 0.633), Jaccard (R^2^ = 0.02 *p* = 0.093), unweighted Unifrac (R^2^ = 0.03 *p* = 0.127) and weighted Unifrac (R^2^ = 0.02 *p* = 0.497).

When analyzing results were according to dominant and recessive allelic grouping (CC vs. TC_TT and TT vs. TC_CC) no significant differences between groups were observed for alpha diversity parameters except for Faith’s Phylogenetic Diversity common allele TT vs. rare allele TC_CC grouping (*p* = 0.006; CC vs. TC_TT *p* = 0.167; Supplementary Figure S1). Other alpha diversity parameters showed no significant variations: observed features (TT vs. TC_CC *p* = 0.105; CC vs. TC_TT *p* = 0.380), Shannon entropy (TT vs. TC_CC *p* = 0.063; CC vs. TC_TT *p* = 0.164), or Pielou evenness (TT vs. TC_CC *p* = 0.144; CC vs. TC_TT *p* = 0.234).

**Figure 2:**
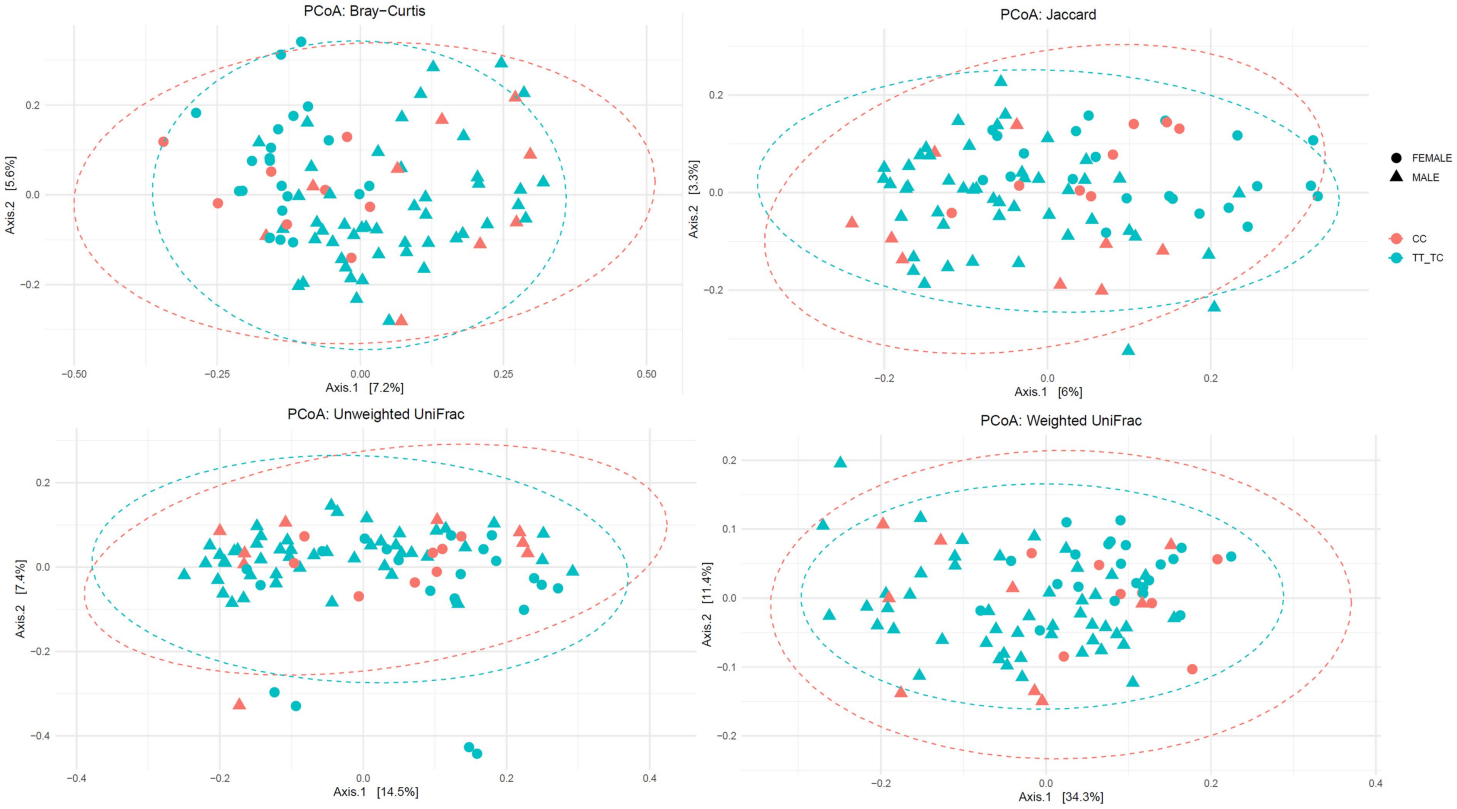
Principal coordinates analysis plots of Bray-Curtis, Jaccard, unweighted and weighted Unifrac distance metrics for VDR polymorphisms according to common versus rare allelic grouping (TT vs. TC_CC).

Additionally, no significant differences were found in β-diversity parameters except for unweighted Unifrac distance metric in the TT vs. TC_CC grouping (Figure 2; Supplementary Figures S2, S3): Jaccard (TT vs. TC_CC: R^2^ = 0.01 *p* = 0.059; CC vs. TC_TT: R^2^ = 0.01 *p* = 0.585), Bray–Curtis (TT vs. TC_CC: R^2^ = 0.01 *p* = 0.181; CC vs. TC_TT: R^2^ = 0.01 *p* = 0.997), unweighted Unifrac (TT vs. TC_CC: R^2^ = 0.02 *p* = 0.026; CC vs. TC_TT: R^2^ = 0.01 *p* = 0.743), and weighted Unifrac (TT vs. TC_CC: R^2^ = 0.01 *p* = 0.572; CC vs. TC_TT: R^2^ = 0.01 *p* = 0.709) distance metrics.

### 3.4 Differential abundance analysis

**Figure 3:**
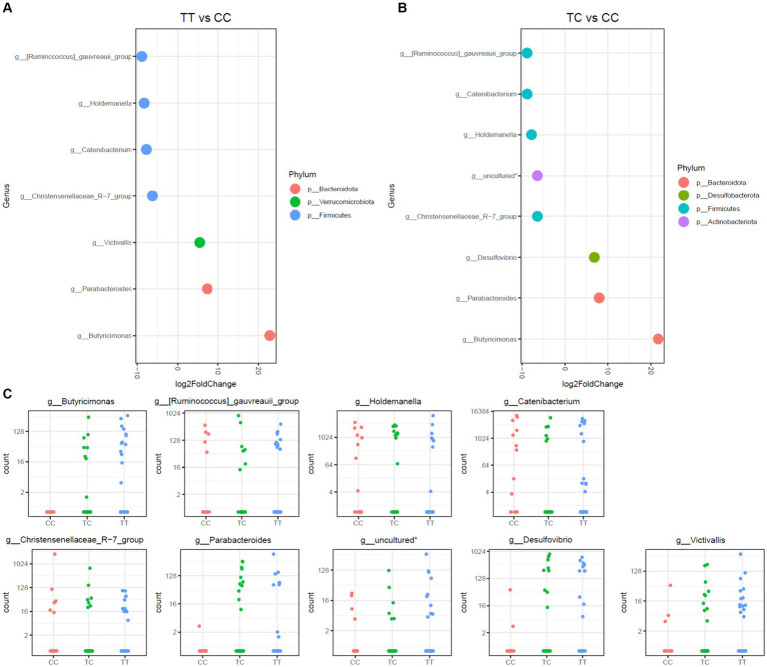
Differential abundance analysis of *VDR* polymorphisms (TT, TC, CC) by DESeq2 adjusted by sex. **(A)**. Log fold changes for all significant genera between TT and CC. **(B)**. Log fold changes for all significant genera between TC and CC. **(C)**. Normalized abundances of genera identified by differential abundance analysis. Boxplots represent normalized count abundances of individual genera in each group (CC, TC, TT). *Belonging to order_Coribacteriales_family_uncultured_genus_uncultured.

**Figure 4:**
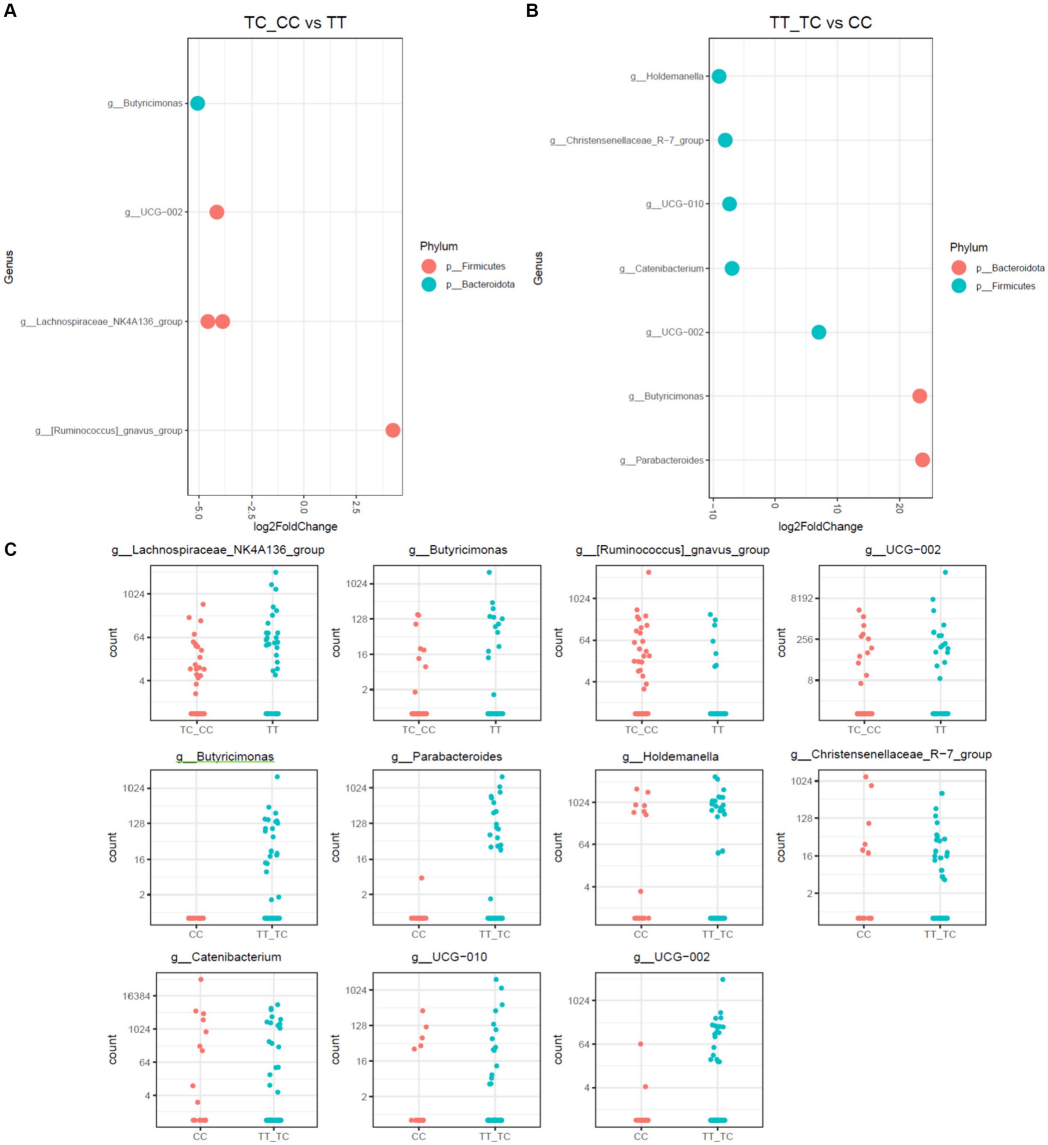
Differential abundance analysis of *VDR* polymorphisms in allelic grouping by DESeq2 adjusted by sex results for DESeq2 adjusted by sex. **(A)** Log fold changes for all significant genera between TC_CC and TT. **(B)** Log fold changes for all significant genera between TT_TC and CC. **(C)** Normalized abundances of genera identified by differential abundance analysis. Boxplots represent normalized count abundances of individual genera in each group.

This study further assessed disparities in microbiota communities using DESeq2 methodology on three distinct polymorphisms, TT, CC, and TC, as depicted in Figures 3, 4. Notably, the CC polymorphism on taxa within the *Parabacteroides* and *Butyricimonas* genera, both constituents of the *Bacteroidota* phylum, and the *Victivallis* genus, showed a decrease when compared to TC and TT polymorphisms. In contrast, genera from *Firmicutes* phylum, inlcuding *Ruminococcus gauvreauii* group, *Holdemanella, Catenibacterium*, and the *Christensenellaceae*_R-7 group, were increased in CC polymorphism, as illustrated in Figure 3. Noteworthily, no significant disparities in microbiota were observed between TC and TT polymorphisms.

When allelic grouping was employed for comparison of microbiota communities [carriers for the common allele (TT) vs. rare allele (CC and TC) and carriers for the recessive allele (CC) vs. dominant allele (TT and TC)], similar outcomes were discerned (Figure 4). The microbiota associated with the CC allelic variant also showed a decrease in *Parabacteroides* and *Butyricimonas*, alongside with an increase in the taxa *Holdemanella, Catenibacterium*, and the *Christensenellaceae*_R-7 group. Additionally, in the comparison of the TC_CC allelic variants, a reduction of *Butyricimonas* and a rise of the *Ruminococcus gauvreauii* group were observed.

**Figure 5:**
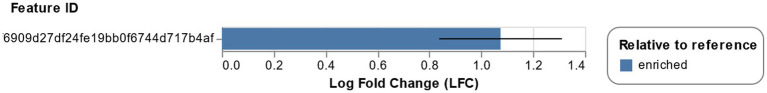
Differential abundance analysis by ANCOM BC adjusted by sex. Log fold change of differentially abundant ASV corresponding to *Butyricimonas virosa* when comparing TT_TC to CC.

ANCOM-BC, adjusted for sex, was employed to investigate dissimilarities in microbial communities. Notably, only a single Amplicon Sequence Variant (ASV) affiliated with *Butyricimonas virosa* (*B. virosa*) exhibited enrichment in the microbiota of the TT_TC group when compared with the CC group, as illustrated in Figure 5. No other taxonomic differences were identified when performing comparisons among genotyping groups (TT, TC, CC) or when comparing allelic groups TC_CC and TT.

### 3.5 Predicted functional metagenome by PICRUSt

**Figure 6:**
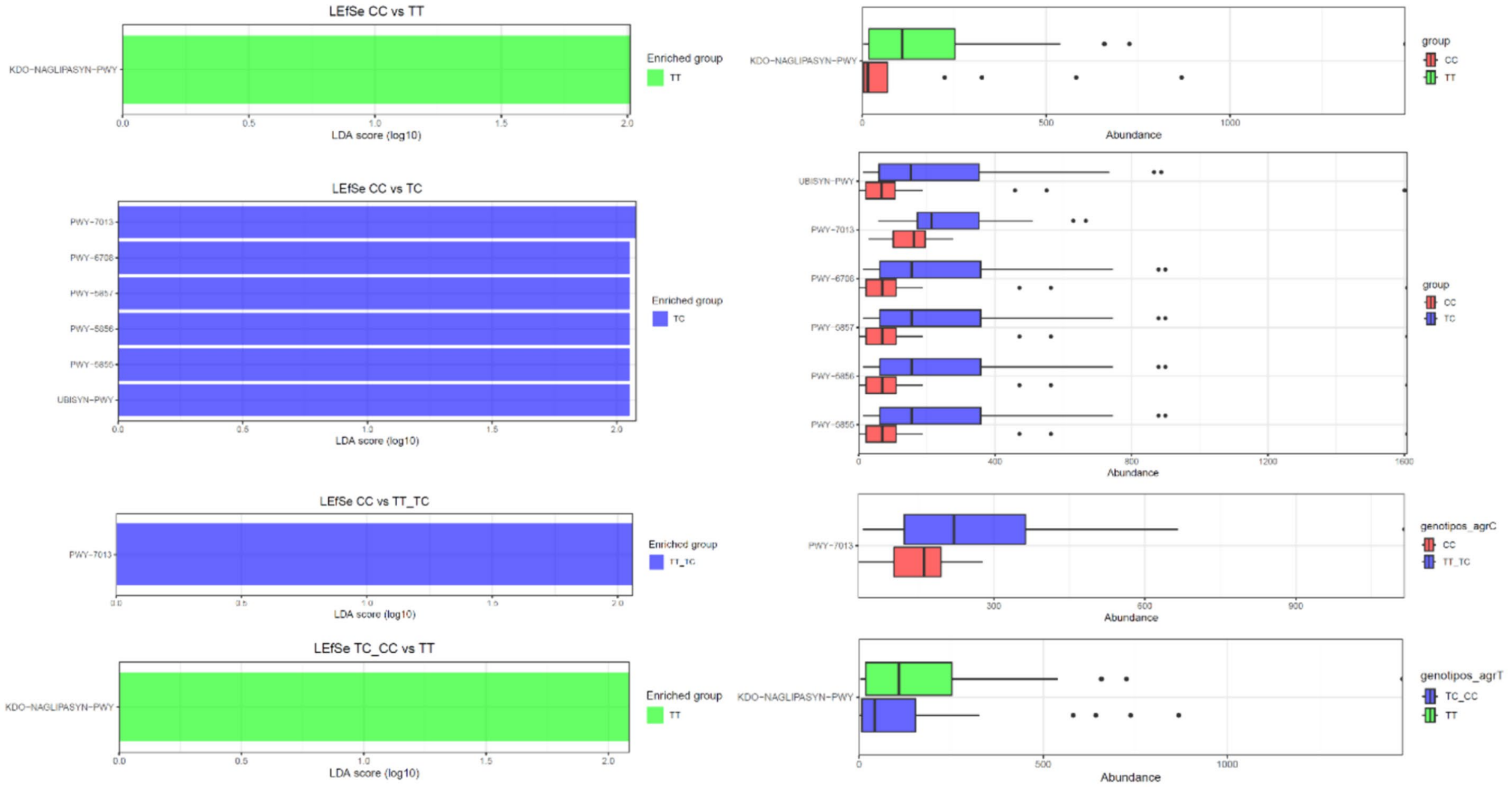
Predicted functional composition of metagenomes based on 16S rRNA gene sequencing data. LEfSe based on the PICRUSTt2 dataset revealed differentially enriched metabolic pathways associated with VDR polymorphisms (TT, TC, CC, TT_TC, TC_CC).

PICRUSt was employed to infer the functional capacities of microbial communities by projecting functional genes linked to various taxa (Figure 6). No significant differences in metabolic pathways were observed when comparing the microbiota from TT and TC polymorphisms. However, LEfSe analysis revealed the enrichment of several pathways within the CC polymorphisms. These pathways included nivalenol biosynthesis (PW 7013), ubiquinol-8 biosynthesis (early decarboxylation; PWY-6708), ubiquinol-10 biosynthesis (early decarboxylation; PWY-5857), ubiquinol-9 biosynthesis (early decarboxylation; PWY-5856), ubiquinol-7 biosynthesis (early decarboxylation; PWY-5855), and the superpathway ubiquinol 8 (UBISYN-PWY) within microbial communities associated with CC polymorphisms when compared to TC. In addition, nivalenol biosynthesis (PW 7013) was the only pathway that exhibited upregulation in CC when contrasted with TT_TC. Furthermore, the superpathway of (Kdo)2-lipid A biosynthesis (KDD-NAGLIPASYN-PWY) displayed downregulation in the CC group when compared with TT, as well as in the TC_CC group when compared to TT.

## 4 Discussion

The genetic variations associated with TaqI rs731236 have been linked to diverse responses in obesity, immune regulation, and inflammatory pathways, suggesting a potential influence on the composition and function of the gut microbiota. This study aimed to characterize the bacterial communities in individuals with different genotypes of the *VDR* gene genotypes (TaqI rs731236), employing the 16S rRNA gene study approach. We investigated microbial diversity and abundance across groups (CC, TT, TC), to explore potential associations between the TaqI polymorphism alleles C/T and genotypes groups with specific microbial profiles.

Analyses of alpha-diversity and beta-diversity reveal that among all the studied indices, only those considering phylogenetic diversity (Unweighted UniFrac, Faith PD) are significantly different among populations with different genotypes for the TaqI polymorphism of the *VDR* gene. This indicates that the observed differences among genotypes are primarily due to variations in the presence of taxonomically distinct bacterial species rather than abundance differences. Different phylogenetic lineages can play specific roles in the functionality of the microbiota (38). Therefore, changes in phylogenetic diversity may impact the metabolic and biological functions of the microbial community. The phylogenetic diversity was found to be higher in individuals with the TT genotype, possibly indicating a distinct and/or enhanced functionality of the microbiota in this group. The TT genotype/T allele is linked to a higher abundance of the *Parabacteroides* and *Butyricimona*s genera, recognized for their roles in degradation of complex carbohydrates and production of short-chain fatty acids. The *Parabacteroides* genus, a commensal member of the gut microbiota, has previously been associated as being influenced by the *VDR* gene (16). Species within *Parabacteroides* act as immune system regulators and possess anti-inflammatory properties (39). Some members of this genus play a crucial role in the digestion of complex carbohydrates, producing short-chain fatty acids (SCFAs) such as acetate, propionate, and butyrate, thereby providing benefits to the host. Hence, modifications in the population of this genus have been associated to host’s health/disease status, with some species suggested as next-generation probiotics (39). The *Butyricimonas* genus, as its name suggests, is associated with butyric acid production, offering several benefits, including energy provision to colonocytes, immune system modulation by providing anti-inflammatory properties, improved nutrient absorption, and hepatic lipid metabolism regulation (40). *B. virosa* has been proposed to have therapeutic potential for preventing high-fat diet (HFD)-induced diabetes and metabolic disorders in mice. Both live and heat-killed *B. virosa* improved body weight, serum glucose level, insulin resistance, and liver steatosis in HFD-induced obese mice (41). Nevertheless, the TT genotype has also been associated to increased insulin resistance and chronic low-grade inflammation (42). This could be explained by the fact that microbiota that produces a high amount of short-chain fatty acids is also associated with microbiota dysbiosis, obesity and cardiometabolic risk. However, the mentioned study does not conduct an analysis of the microbiota, and currently, this is the first time in the literature that the TT genotype/T allele is associated with the presence of *Butyricimonas* in the microbiota (43). Further research in this area is needed.

Finally, the *Victivallis* genus, overrepresented in the TT genotype/ T allele, is a sugar fermenter and acetate producer (44). *Victivallis*, known for anaerobic sugar fermentation, alongside genera like *Parabacteroides* and *Butyricimonas*, known for complex carbohydrate degradation and butyrate production, respectively, are also significant components of the gut microbiota. *Victivallis* has been associated with decreased adiposity, hepatic steatosis, and diabetes in animal models (45, 46), and is less abundant in hypercholesterolemic patients (47), suggesting potential positive effects on glucose and fat metabolism in individuals with the TT genotype/ T allele.

Conversely, groups less represented in the TT genotype included the *R. gauvreauii* group, *Catenibacterium, Holdemanella*, and the *Christensenellaceae*_R-7 group. The *R. gauvreauii* group is a bile-resistant acetate acid producer using sucrose and sugar alcohols as carbon sources (48). *R. gauvreauii*, stands out for its inability to metabolize resistant starches but proficient growth on sugar alcohols (49). This unique metabolic behavior may have implications for substrate utilization within the gut. It has been observed that *R. gauvreauii* increases in gut microbiota of obese men post exercise and is positively associated with respiratory capacity and negatively with visceral fat (50). *Catenibacterium*, a member of the Christensenellaceae family, has been linked to leanness and lower BMI, suggesting roles in energy metabolism and dietary fiber digestion. Its species produce acetic, lactic, butyric, and isobutyric acids from sugars (51). However, its health impacts are not fully unclear. While reduced *Catenibacterium* abundance has been linked to obesity (52), conflicting evidence showed its enrichment in obesity cases (53). High *Catenibacterium* levels have also been connected to lower trimethylamine oxide (TMAO) levels, a cardiovascular disease-related metabolite (54). *Holdemanella*, a relatively unknown genus, has been investigated for potential roles in fermentation and anti-inflammatory properties, particularly in colitis contexts. It is involved in carbohydrate metabolism and associated with health benefits due to its anti-inflammatory properties (55) and regulatory effects on glucose metabolism (56). However, links to pathological states such as thyroid cancer have been observed (57). Different *Holdemanella* taxa have been positively associated with android-type obesity in men and negatively in women (58). Lastly, the *Christensenellaceae*_R7 group, more prevalent in the CC genotype, is a heritable microbiota component (59) associated with metabolic health in children (60) and adults (61). It has also been linked to lower visceral fat and lean mass in several studies (62–64).

In the comparative analysis of metabolic profiles between genotypes CC and TT, the TT group showed an overexpression of the KDD-NAGLIPASYN-PWY pathway. This pathway is involved in the biosynthesis of lipid A, a key component of lipopolysaccharides (LPS) present in Gram-negative bacteria’s the outer membrane. This suggests a higher presence of Gram-negative bacteria or a specific immunological response in the TT group (16). When analyzing metabolic differences between genotypes CC and TC, pathways related to coenzyme Q (CoQ) biosynthesis, particularly the UBISYN-PWY pathway, were associated with the TC group. Ubiquinol-8, essential for the electron transport chain and cellular energy production, was significantly overexpressed in the TC group, indicating potential implications in metabolism and inflammation (65). In the comparison of CC vs. TT_TC genotypes, CoQ biosynthesis pathways (PWY-6708, PWY-5857, and PWY-5856) were also prominent in the TC group, emphasizing the importance of CoQ biosynthesis in this context. Furthermore, the TT_TC group showed overexpression of the PWY-7013 pathway (nivalenol biosynthesis and propane 1,2 diol degradation) indicating an increased degradation of this compound, potentially affecting energy metabolism or substrate utilization. In the TC_CC vs. TT comparison, pathways associated with menaquinone biosynthesis (PWY-5853) were also overexpressed in the TC group. Menaquinones, types of vitamin K produced by certain bacteria, are involved in electron transport. These observed differences may reflect alterations in the presence or metabolic activity of vitamin K-producing bacteria.

The results reveal substantial differences in metabolic pathways among the studied groups (CC, TT, TC, TT_TC), emphasizing the significance of lipid A and CoQ biosynthesis, as along with changes in the degradation of specific compounds and the biosynthesis of menaquinones. These findings suggest potential associations with specific VDR gene profiles.

Polymorphisms in the VDR gene can lead to significant differences in gut microbiota composition and diversity through several key mechanisms. The VDR gene encodes the vitamin D receptor, which regulates the immune system by influencing the expression of antimicrobial peptides that maintain the gut barrier and control pathogenic bacteria (66). VDR polymorphisms can alter immune responses, affecting gut inflammation levels and creating environments that favor certain microbial communities. Additionally, the VDR gene is expressed in gut epithelial cells, where it regulates cell proliferation, differentiation, and apoptosis processes vital for maintaining gut barrier integrity (5). Disruptions in VDR activity can influence which microbial species thrive, as evidenced by VDR knockout mice exhibiting distinct microbial profiles (20, 67). Furthermore, VDR polymorphisms may affect vitamin D status, impacting nutrient absorption and metabolism, which in turn alters the gut environment and influences microbial growth (68).

The VDR gene TaqI polymorphism can directly affect the composition and functionality of the gut microbiome due to the critical role that vitamin D signaling plays in immune regulation and gut health. Changes in microbiota due to differences in the TaqI genotypes could be related to different gene transcription associated with the genotypes. However, there are inconsistent results regarding which genotypes produce higher levels of mRNA expression, although there appears to be a general trend indicating that the C allele demonstrates a more favorable response than the T allele (7).

The TT genotype of the TaqI polymorphism may be associated with differential modulation in vitamin D levels or VDR activity which could favor an environment that benefits certain bacterial genera. This could be an environment that promotes the production or utilization of SCFAs, thereby benefiting the proliferation of bacteria involved in butyrate production and other SCFAs such as *Butyricimonas* and *Parabacteroides*. VDR is also involved in the regulation of bile acid metabolism. Thus, alterations in VDR signaling due to the TT genotype could modify bile acid composition in the gut, facilitating the growth of genera like Parabacteroides, with capability to metabolize bile acids. Moreover, for the TT genotype, there could be differential modulation of cytokines and other immunomodulatory molecules, creating an intestinal environment that favors the proliferation of specific bacteria such as *Butyricimonas* and *Parabacteroides*.

Overall, VDR gene polymorphisms can lead to altered vitamin D signaling, which impacts immune regulation, gut barrier integrity, and the gut environment, ultimately influencing the composition and diversity of the gut microbiome. These changes can have significant consequences for host health, including impacts on immune function, inflammation, nutrient absorption, and susceptibility to various diseases.

A limitation of the present study is that although we used a well-defined sample of healthy participants, the study had a relatively small sample size, which may limit the generalizability of the findings to larger populations. However, studying the association between genetic variants and the microbiota in healthy volunteers facilitates the examination of the direct influence of genetic variants on microbiota composition without confounding pathological factors. Moreover, in our study potential confounding factors, such as sex, dietary habits and smoking has been considered.

## 5 Conclusion

Our research work suggests that polymorphisms in the VDR gene may be linked to specific microbiota genera compositions and functionalities. This study highlights the importance of considering genetic variations, like VDR gene polymorphisms, to grasp the intricate interplay between host genetics and gut microbiota, which may have implications for personalized nutrition and health interventions. Further research is essential to unravel the precise mechanisms by which VDR gene polymorphisms influence gut microbiota composition and function, thereby setting the stage for future investigations and potential therapeutic approaches.

Knowledge of how VDR polymorphisms impact gut microbiota can contribute to the development of personalized medical approaches. Individuals with certain VDR gene variants might benefit from tailored probiotic or dietary interventions to optimize gut health.

## Data Availability

The data presented in the study are deposited in the NCBI BioProject repository, accession number PRJNA1152925.

## Footnotes

^1^https://github.com/BenKaehler/readytowear
