# Supplementary material for: VDR gene TaqI (rs731236) polymorphism affects gut microbiota diversity and composition in a Caucasian population
Source: Front Nutr. 2024 Sep 12;11:1423472. doi: 10.3389/fnut.2024.1423472 (PMC11425793; doi:10.3389/fnut.2024.1423472)
Supplement: Supplementary file 1 [file Data_Sheet_1.pdf]

SUPPLEMENTARY MATERIAL

Table S1. Age, sex, and body composition parameters of participants according to their genotypes

	TT	TC	CC	p[a]	TC_CC	p[b]	TT_TC	p[c]
Sex (n/%)	26/70.3M<br>11/29.7W	22/66.7M<br>11/33.3W	9/52.9M<br>8/47.1W	0.454*	31/35.6M<br>19/64.4W	0.422*	48/55.2M<br>22/44.8W	0.224*
Age (years)	32 (13.00)	36 (9.00)	37 (5.00)	0.824	36 (9.00)	0.638	36.00 (12.00)	0.864
Height (m)	171.63±8.88	173.58±1.37	168.23±8.53	0.449	172.07±7.91	0.979	172.54 ±8.16	0.240
BM (kg)	68.24±12.32	71.23±1.49	66.47±10.01	0.409	69.89 ±8.68	0.709	69.64±10.51	0.314
BMI (kg/m^2)	22.50 (4.07)	23.40 (2.48)	24.60 (4.00)	0.781	23.50 (3.24)	0.613	23.14 (3.04)	0.813
BFP (%)	22.80 (12.21)	21.45 (10.80)	24.50 (17.70)	0.906	21.60 (14.50)	0.700	22.10 (11.15)	0.968
BFM (Kg)	15.26 (9.17)	14.37 (7.60)	16.03 (8.23)	0.824	14.68 (7.07)	0.534	14.76 (8.12)	0.804
VAT (g)	270.00 (235.80)	273.00 (137.80)	281.00 (210.00)	0.950	281.00 (142.00)	0.952	270.00 (191.00)	0.794

BM: body mass; BMI: body mass index; BFP: body fat percentage; BFM: body fat mass; VAT: estimated visceral fat. [a]: Genotypic comparison TT vs TC vs CC. [b]Allelic comparison TT vs TT_CC. [c]Allelic comparison CC vs TT_TC. Values are mean ± standard deviation or median and interquartile range. *Chi-squared test

Table 2. Total energy, macronutrients, and fiber dietary intake

	TT	TC	CC	p[a]	TC_CC	p[b]	TT_TC	p[c]
Energy (kcal/day)	2204.10 (839.50)	1947.15 (867.58)	1808.70 (966.40)	0.493	1918.61 (712.70)	0.483	2098.25 (854.3)	0.244
CHO*	42.50 (8.00)	43.00 (8.00)	45.00 (12.00)	0.577	43.00 (9.00)	0.971	43.00 (8.00)	0.349
Protein*	18.00 (4.00)	18.00 (6.00)	16.00 (7.00)	0.980	17.00 (5.00)	0.911	18.00 (4.00)	0.843
Fat*	40.00 (10.00)	39.00 (11.00)	39.00 (7.00))	0.846	39.00 (7.00)	0.934	39.00 (10.00)	0.627
Protein/CHO	0.38 (0.12)	0.40 (0.12)	0.35 (0.21)	0.607	0.39 (0.15)	0.927	0.39 (0.13)	0.387
Protein/fat	0.47 (0.16)	0.45 (0.21)	0.41 (0.49)	0.851	0.44 (0.24)	0.818	0.46 (0.19)	0.708
Fiber (g/day)	26.55 (15.80)	20.35 (20.13)	18.2 (21.1)	0.411	18.60 (18.60)	0.293	23.40 (17.05)	0.237

CHO: carbohydrates; *Percentage of energy. Values are median and interquartile range. [a]Genoytpic comparison TT vs TC vs CC. [b]Allelic comparison TT vs TT_CC. [c]Allelic comparison CC vs TT_TC.

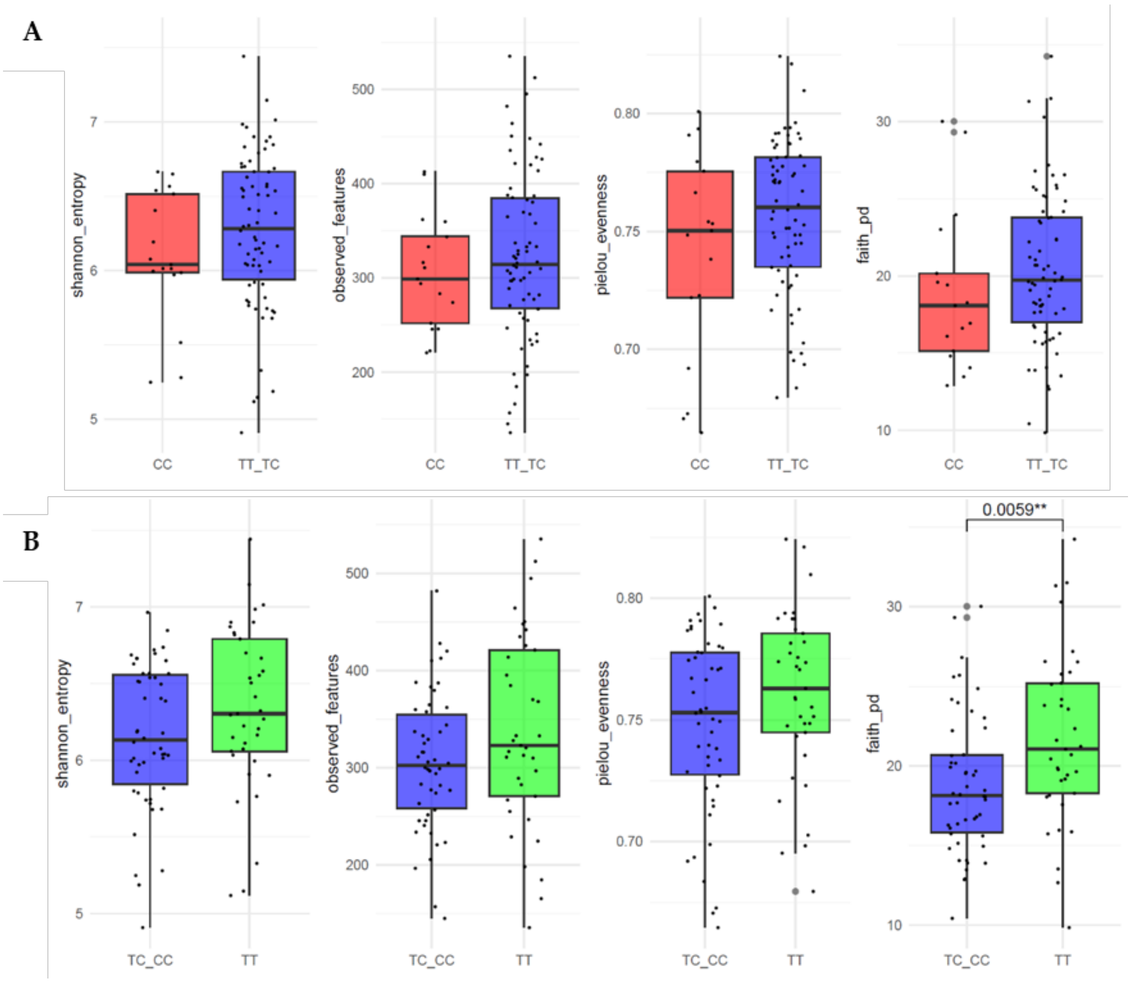

Figure S1. Alpha diversity indices of *VDR* polymorphisms in allelic grouping: Shannon entropy, Observed features, Pielou evenness and Faith's Phylogenetic Diversity. (**A)**: TT vs TC_CC; (**B**): CC vs TC_TT.

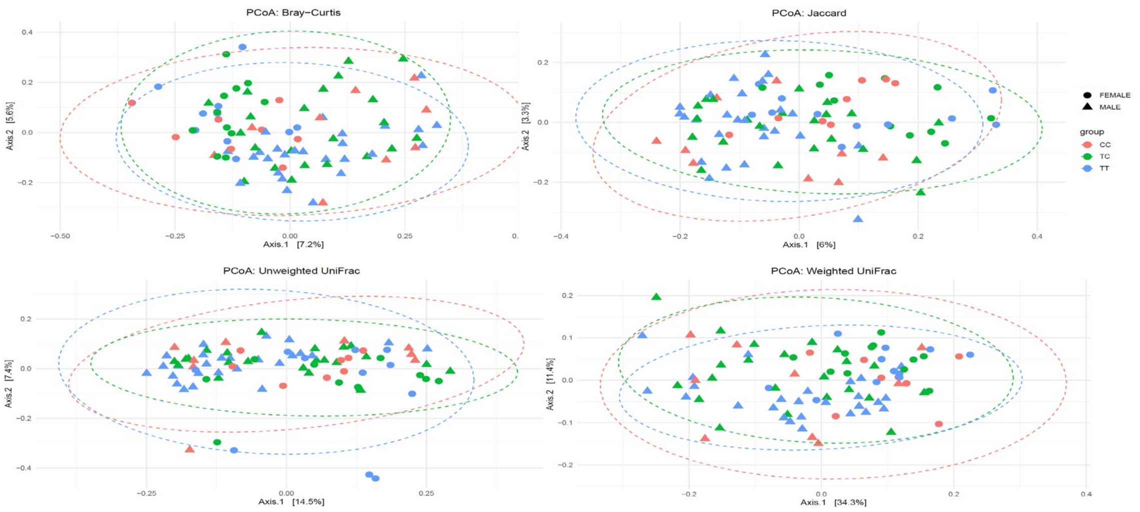

Figure S2. Principal coordinates analysis plots of Bray-Curtis, Jaccard, unweighted and weighted Unifrac distance metrics for VDR polymorphisms (CC, TC, TC).

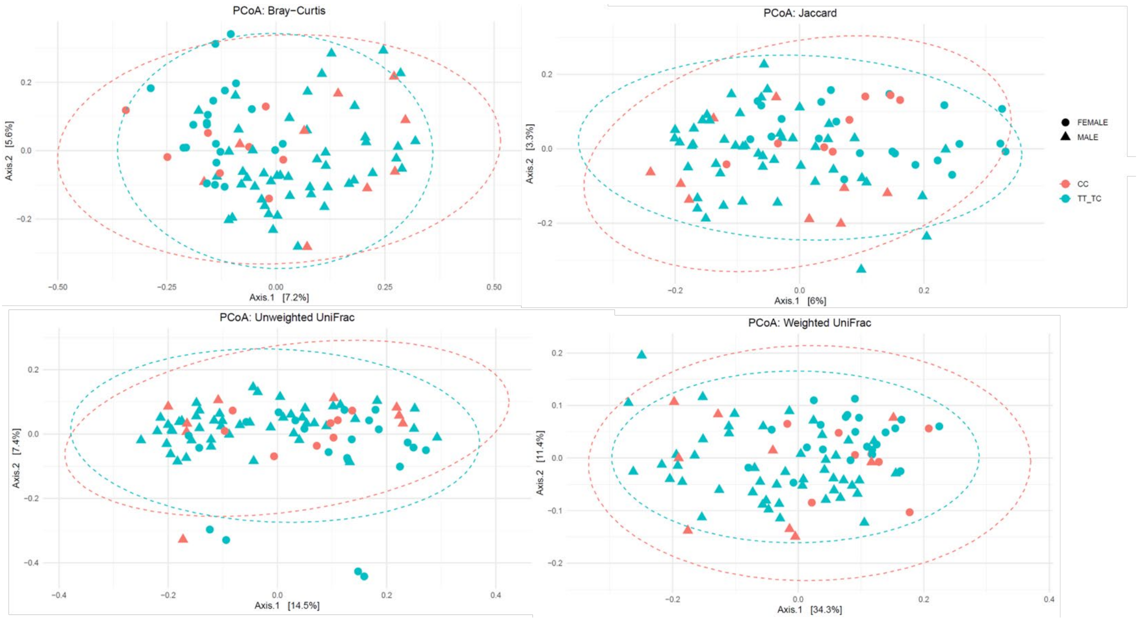

Figure S3. Principal coordinates analysis plots of Bray-Curtis, Jaccard, unweighted and weighted Unifrac distance metrics for VDR polymorphisms according to common versus rare allelic grouping (CC vs TT_TC).
